# Radiation-Induced Pneumonitis in the Era of the COVID-19 Pandemic: Artificial Intelligence for Differential Diagnosis

**DOI:** 10.3390/cancers13081960

**Published:** 2021-04-19

**Authors:** Francesco Maria Giordano, Edy Ippolito, Carlo Cosimo Quattrocchi, Carlo Greco, Carlo Augusto Mallio, Bianca Santo, Pasquale D’Alessio, Pierfilippo Crucitti, Michele Fiore, Bruno Beomonte Zobel, Rolando Maria D’Angelillo, Sara Ramella

**Affiliations:** 1 Departmental Faculty of Medicine and Surgery, Diagnostic Imaging and Interventional Radiology, Università Campus Bio-Medico di Roma, 00128 Rome, Italy; f.giordano@unicampus.it (F.M.G.); c.mallio@unicampus.it (C.A.M.); p.dalessio@unicampus.it (P.D.); b.zobel@unicampus.it (B.B.Z.); 2 Departmental Faculty of Medicine and Surgery, Radiation Oncology, Università Campus Bio-Medico di Roma, 00128 Rome, Italy; e.ippolito@unicampus.it (E.I.); c.greco@unicampus.it (C.G.); b.santo@unicampus.it (B.S.); m.fiore@unicampus.it (M.F.); s.ramella@unicampus.it (S.R.); 3 Departmental Faculty of Medicine and Surgery, Thoracic Surgery, Università Campus Bio-Medico di Roma, 00128 Rome, Italy; p.crucitti@unicampus.it; 4 Departmental Faculty of Medicine and Surgery, Radiation Oncology, Università degli Studi Tor Vergata, 00133 Rome, Italy; profrmdangelillo@gmail.com

**Keywords:** COVID-19, radiation pneumonitis, artificial intelligence, deep learning, chest CT

## Abstract

**Simple Summary:**

Radiation-induced pneumonitis and severe acute respiratory syndrome coronavirus 2 (SARS-CoV-2) interstitial pneumonia show overlapping clinical features. As we are facing the COVID-19 pandemic, the discrimination between these two entities is of paramount importance. In fact, lung cancer patients are at higher risk of complications from SARS-CoV-2. In this study, we aimed to investigate if a deep learning algorithm was able to discriminate between COVID-19 and radiation therapy-related pneumonitis (RP). The algorithm showed high sensitivity but low specificity in the detection of RP against COVID-19 pneumonia (sensitivity = 97.0%, specificity = 2%, area under the curve (AUC = 0.72). The specificity increased when an estimated COVID-19 risk probability cut-off of 30% was applied (sensitivity 76%, specificity 63%, AUC = 0.84).

**Abstract:**

(1) Aim: To test the performance of a deep learning algorithm in discriminating radiation therapy-related pneumonitis (RP) from COVID-19 pneumonia. (2) Methods: In this retrospective study, we enrolled three groups of subjects: pneumonia-free (control group), COVID-19 pneumonia and RP patients. CT images were analyzed by mean of an artificial intelligence (AI) algorithm based on a novel deep convolutional neural network structure. The cut-off value of risk probability of COVID-19 was 30%; values higher than 30% were classified as COVID-19 High Risk, and values below 30% as COVID-19 Low Risk. The statistical analysis included the Mann–Whitney U test (significance threshold at *p* < 0.05) and receiver operating characteristic (ROC) curve, with fitting performed using the maximum likelihood fit of a binormal model. (3) Results: Most patients presenting RP (66.7%) were classified by the algorithm as COVID-19 Low Risk. The algorithm showed high sensitivity but low specificity in the detection of RP against COVID-19 pneumonia (sensitivity = 97.0%, specificity = 2%, area under the curve (AUC = 0.72). The specificity increased when an estimated COVID-19 risk probability cut-off of 30% was applied (sensitivity 76%, specificity 63%, AUC = 0.84). (4) Conclusions: The deep learning algorithm was able to discriminate RP from COVID-19 pneumonia, classifying most RP cases as COVID-19 Low Risk.

## 1. Introduction

Radiotherapy plays a key role in the treatment of lung cancer, both as a radical treatment in inoperable patients as well as induction therapy or adjuvant treatment for resectable disease [1,2,3].

The mechanism of radiation-induced lung injury (RILI) depends on direct DNA damage and production of reactive oxygen species. The latter causes cell loss, edema of the alveolar walls and enhanced vascular permeability, leading to the apoptosis of alveolar type-1 pneumocytes. After apoptosis, cells start recruiting immune effector cells that activate tissue remodeling [4]. The clinical picture of RILI is radiation pneumonitis (RP). Symptomatic RP occurs in about 30% of patients receiving concurrent chemoradiation (CCRT) for non-small cell lung cancer (NSCLC) [5]. The incidence of RP is multifactorial, including treatment-related and patient-related conditions, particularly older age and larger volume of lungs receiving higher doses; some chemotherapy regimens are also associated with increased risk of RP [5].

In 2019, the severe acute respiratory syndrome coronavirus 2 (SARS-CoV-2) was identified in Wuhan, China. As the epidemic rapidly spread around the world, in March 2020, the World Health Organization (WHO) declared a global public health emergency, describing the situation as a pandemic [6]. The respiratory syndrome associated with SARS-CoV-2 includes variable degrees of severity. The most serious clinical entity is a severe interstitial pneumonia that can lead to acute respiratory distress [7].

RP and SARS-CoV-2 interstitial pneumonia show overlapping clinical features. In fact, the most common RP symptoms are dyspnea, dry non-productive cough and fever. In addition, most patients show a high erythrocyte sedimentation rate or C-reactive protein with normal serum procalcitonin and high serum ferritin and D-Dimer as expressions of cancer disease. Moreover, lymphopenia is frequently observed, as lymphocytes are more radiosensitive than other white blood cells. Finally, chest CT findings are also very similar, as the radiological characteristics of RP are ground-glass opacity (GGO) in the initial phase, patchy areas of consolidation in the peak phase and fibrotic changes in the dissipative phase [8].

As we are moving from the peak of the pandemic to the longer mitigation phase, the discrimination between these two entities is of paramount importance [9]. In fact, lung cancer patients are more fragile and are at higher risk of complications from SARS-CoV-2 due to their being immunocompromised and reduced lung function [10].

Various radiological techniques may be used for the detection and quantification of lung involvement in COVID-19 [11,12], but CT proved to be valuable for both purposes [13,14,15,16].

Deep learning (DL), a form of artificial intelligence (AI), is becoming a promising support for medical imaging due to its capability of feature extraction and analysis [17,18,19,20]. It has been successfully applied to chest CT imaging to distinguish COVID-19 pneumonia cases from community-acquired infections [21] as well as to provide qualitative and quantitative analyses for disease burden estimation, facilitating and expediting imaging interpretation [22,23].

Based on the above considerations, the aim of the present study was to test the performance of a deep learning algorithm in discriminating RP from COVID-19 pneumonia.

## 2. Materials and Methods

We designed a retrospective observational study, performed in accordance with the Declaration of Helsinki. The local Ethical Committee approved the study (the ethic code is Prot.: 88/20 OSS.NOT ComEt CBM, 06 October 2020) and waived the need for written informed consent from participants.

### 2.1. Participants

All of the subjects underwent chest CT scan and were consecutively sampled from our electronic database. In this study, three groups of patients were included and classified according to both medical history and CT imaging findings (i.e., radiological reports). The patients were grouped as follows.

Group 1 (control group): A group of consecutive symptomatic (fever > 37.5 °C, dyspnea and/or cough and/or fatigue) patients with negative chest CT scan acquired between 15 March 2020 and 30 April 2020.

Group 2: A group of consecutive symptomatic (fever > 37.5 °C, dyspnea and/or cough and/or fatigue) patients with confirmed COVID-19 pneumonia by positive RT-PCR (RealTiMe SARS-CoV-2 Assay, Abbott Laboratories. Abbott Park, IL, USA) on a nasopharyngeal (or oropharyngeal) specimen using the swab technique and positive chest CT scan acquired between 15 March 2020 and 30 April 2020.

Group 3: A group of consecutive patients with lung cancer treated with chemoradiation or radiotherapy alone and a positive history of RP confirmed by chest CT scan acquired from 2015 up to September 2019, before the occurrence of any known case of COVID-19 in Italy.

Exclusion criteria were patients with history of previous radiation therapy on the thoracic region for other pathologies (such as breast cancer) and poor quality of CT images as well as insufficient chest expansion or movement artefacts.

In Group 1 and Group 2, lung CT scans were indicated to explore indeterminate findings on chest X-ray and/or to quantify lung involvement in symptomatic patients. In Group 3, CT images were taken from oncological follow-up whole-body CT scans and retrospectively analyzed.

### 2.2. Chest CT Imaging Protocol

All chest CT acquisitions were obtained by maintaining the patients in a supine position during end-inspiration, with or without contrast medium injection. Chest CT images were acquired using either a Dual Source 384-slice (2 × 192) CT (Siemens SOMATOM Force, Erlangen, Germany; tube real-time voltage modulation, 70–150 kV; tube real-time dose modulation (CARE Dose4D™), 80–250 mAs; spiral pitch factor, 1.8; collimation width, 0.6 mm), a 128-slice CT (Siemens SOMATOM Definition AS, Erlangen, Germany; tube voltage, 120 kV; tube real-time dose modulation (CARE Dose4D™), 80–250 mAs; spiral pitch factor, 1.2; collimation width, 0.6 mm) or a 40-slice CT (Philips Brilliance CT; tube voltage, 120 kV; 80–150 mAs; spiral pitch factor, 1.0; collimation width, 0.625 mm).

Every patient’s temperature was taken before entering the hospital, and dedicated paths were established for oncologic patients. Moreover, all patients and medical staff wore the proper personal protective equipment (PPE) during hospital stay and during CT scan acquisition, and a meticulous decontamination of the CT room and passive air exchange was carried out after every scan performed on patients with clinical or imaging suspicion of COVID-19.

### 2.3. Deep Learning Algorithm Analysis

The deep learning algorithm analysis was performed using InferRead^TM^ CT Lung (COVID-19) (Infervision, Europe GmbH, Wiesbaden, Germany), an AI solution specifically developed for diagnosis and management support of COVID-19 pneumonia. Among its features, the algorithm module includes automated segmentation of the core features of COVID-19 lung lesions and the segmentation of the lung lobes (right upper lobe, middle lobe, right lower lobe, left upper lobe and left lower lobe). The output also includes the estimated risk probability for the diagnosis of COVID-19 pneumonia. The core algorithm is based on a novel deep convolutional neural network structure and uses the U-net network structure as the core segmentation network [24]. The model training process is presented elsewhere [25]. The cleaned and labeled data were trained through the designated network structure. Continuous testing and parameter adjustments resulted in a final model that meets all the requirements. The model was developed initially after training on a population of patients diagnosed in Wuhan, China, and was later further developed through training on a larger population. Specifically, for the trained AI model, patients’ characteristics (*n* = 2191 adult patients; Wuhan Chinese COVID-19) were mixed, including all stages and clinical presentations of the disease (e.g., symptoms could have been mild, moderate or severe) [24]. Based on a preliminary analysis of the deep learning algorithm, evaluated in our hospital with the first 100 patients undergoing RT-PCR test, the cut-off value of the estimated risk probability of COVID-19 was set at levels higher than 30% (“COVID-19 High Risk”), as the percentage of patients confirmed to have COVID-19 above this cut-off value was higher than 95%. Values of estimated risk probability below 30% were classified as “COVID-19 Low Risk” [26], as the definite diagnosis requires a positive RT-PCR test.

**Figure 1:**
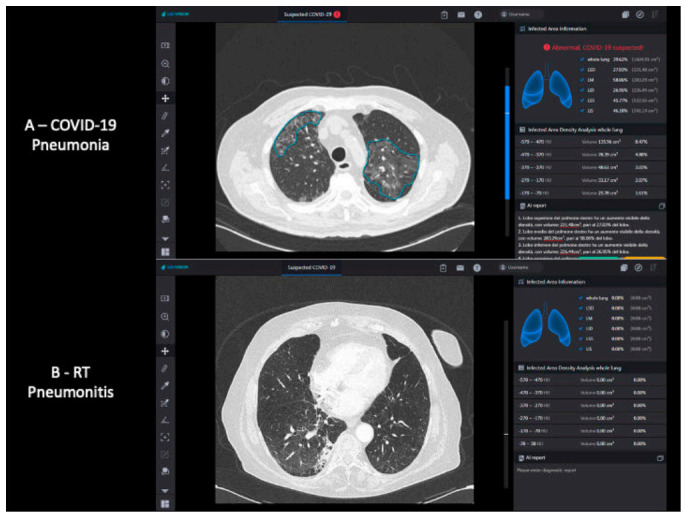
InferRead^TM^ CT Lung (COVID-19) system interface example; comparison between a patient with COVID-19 pneumonia (**A**) and a patient with RP (**B**). COVID-19 = coronavirus disease 2019; RP = radiation-therapy related pneumonitis.

The deep learning processing time for one CT exam is around 10–20 s in a dedicated server with the following configuration characteristics: 16GB RAM, 3TB Drive, graphics processing unit (GPU)-powered Linux server system. The chest CT studies are automatically forwarded to the AI server located on the premises. Once the server receives a study, the AI application starts processing, and the results are stored until a physician assesses them. Two series can be analyzed in parallel given the number of GPU instances available. The vendor agnostic AI system is capable of analyzing CT images generated by different CT machine vendors. The system is able to accept CT images generated by CT machines in different reconstruction protocols with a reconstruction slice thickness lower than 1.5 mm [24]. The result can be also accessed through a URL to the case worklist. An instant alert is received on the case worklist page once the chest CT arrives in the AI server and is deemed as COVID-19 suspicious by the AI application. Figure 1 shows exemplary screenshots of the AI viewer after the assessment of a patient with a confirmed diagnosis of COVID-19 and another with a clinical diagnosis of RP.

### 2.4. Statistical Analysis

Descriptive statistics, including means, medians, ranges and percentiles, were calculated to understand the core tendencies of the enrolled cohorts. Data distribution normality was checked by means of the Kolmogorov–Smirnov test. The Kruskal–Wallis and chi square tests were used to compare age and sex distribution among the groups, respectively.

The primary objective was to assess if the AI quantitative imaging analysis resulted in a statistically significant difference between COVID-19 pneumonia and RP. To investigate this primary outcome, disease risk as well as affected lobes percentages and volumes were compared between Group 2 and Group 3 by using the Mann–Whitney U test. We compared the groups using the chi square test, with a disease risk cut-off value of 30%.

Statistical Package for the Social Sciences (SPSS) software version 26.0 (IBM, Segrate, Milan, Italy) was applied for all aforementioned statistical computations. Additionally, we performed receiver operating characteristic (ROC) curve fitting by using the maximum likelihood fit of a binormal model and calculated the area under the curve (AUC) with the 95% confidence interval (95% CI). Sensitivity, specificity, positive predictive value and negative predictive value are presented as point estimates (95% CI).

## 3. Results

### 3.1. Study Population Characteristics

Table 1 shows the study population characteristics and the three independent datasets testing the AI model. Patients with COVID-19 and RP were older than pneumonia-free patients (*p* = 0.001). The number of males was higher than that of females across all patient groups. A positive SARS-CoV-2 RT-PCR test was available for all patients with COVID-19 and a negative RT-PCR test was available for 14/30 (47%) pneumonia-free patients. In Group 2, chest CT scans showed the known features of COVID-19 pneumonia, including peripheral, bilateral and multi-lobar ground-glass opacity, with or without crazy paving pattern and, in some cases, with consolidations. These features were automatically detected and segmented by the AI software. In Group 3, despite similar CT findings, although mostly unilateral, the diagnosis of RT-related pneumonitis in patients occurred as a complication during the course of and within the first 3 months after radiation treatment. Non-small cell lung cancer (NSCLC) was the most prevalent primary tumor (32/4; 89%) within Group 3. Most patients presenting RP (66.7%) were classified by the algorithm as “COVID-19 Low Risk”. All RP cases classified as “COVID-19 High Risk” were ≥G3 (CTC AE vers. 4.0).

### 3.2. Deep Learning Algorithm Performance

Table 2 shows the performance of the deep learning algorithm on the risk estimation of COVID-19 pneumonia on chest CT images. The sensitivity for discriminating COVID-19 pneumonia from RP was 97% (true positive = 33/34), with 95% CI (0.94–0.99); however, the specificity was very low (2%; 95% CI = 0.0–0.05%) without a COVID-19 risk cut-off value. Indeed, the specificity increased to 63% (95% CI = 0.51–0.74) when the risk cut-off value was set at 30%. The deep learning algorithm’s performance in detecting COVID-19 pneumonia compared with pneumonia-free patients had high sensitivity and specificity (97% and 47%, respectively).

**Figure 2:**
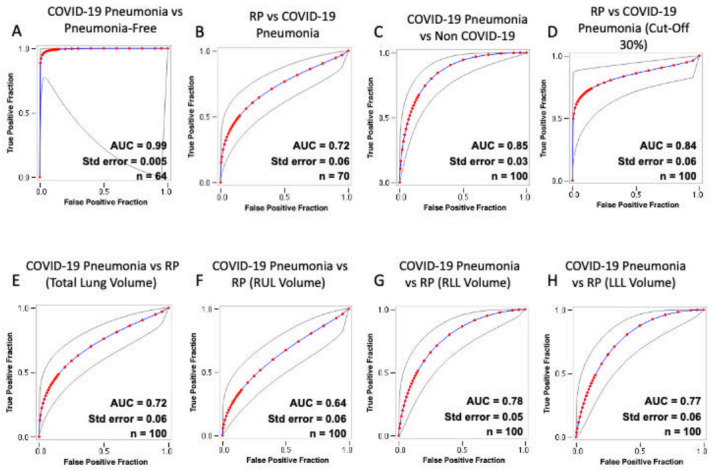
Receiver operating characteristic (ROC) curves of the diagnostic performance of the artificial intelligence (AI) prediction risk of COVID-19 pneumonia. Each plot shows the ROC curve obtained from the testing after including the following pairs: COVID-19 and pneumonia-free patients (**A**); RP and COVID-19 (**B**); COVID-19 and non-COVID-19 patients (**C**); RP and COVID-19 with 30% threshold (**D**); COVID-19 and RP total lung volume involvement (**E**); COVID-19 and RP RUL involvement (**F**); COVID-19 and RP RLL involvement (**G**); COVID-19 and RP LLL involvement (**H**). Gray lines plot 95% confidence intervals. COVID-19 = coronavirus disease 2019; RP = radiation therapy-related pneumonitis; RUL = right upper lobe; ML = middle lobe; RLL = right lower lobe; LUL = left upper lobe; LLL = left lower lobe; AUC = area under the ROC curve; Std. Error = standard error.

The ROC curves for the AI risk prediction of COVID-19 pneumonia are shown in Figure 2. The corresponding AUC value for COVID-19 and RP independent of the 30% cut-off value was 0.72 (95%CI: 0.66–0.78) but increased to 0.84 (95% CI: 0.78–0.90) when applying the 30% risk cut-off value. The AUC values were 0.99 (95% CI: 0.98–1.00) for COVID-19 and pneumonia-free and 0.85 (95% CI: 0.82–0.88) for COVID-19 and non-COVID-19.

Table 3 summarizes the results of the comparison between COVID-19 pneumonia and RP in terms of total and lobar involvement. The total lung volume (*p* = 0.001) and both of the lower lobes’ volumes (*p* < 0.001) were significantly more affected in the COVID-19 group compared to the RP group.

## 4. Discussion

To the best of our knowledge, this is the first study investigating the performance of an AI software in differentiating COVID-19 pneumonia from radiation therapy-related pneumonitis. We used this deep learning algorithm, initially trained on a sample population in Wuhan, China, on an Italian sample population with lung cancer. The deep learning algorithm was able to differentiate COVID-19 pneumonia and RP with good diagnostic performance (AUC 0.72). However, the specificity was very low (2%); indeed, almost all patients with RP were classified as suspected patients with COVID-19 pneumonia, confirming that lung interstitial disease in patients with previous radiation treatment represents a confounding factor in the differential diagnosis of COVID-19 pneumonia. Nevertheless, the assessment of CT scans based on the low-risk/high-risk classification that used the 30% cut-off value showed a net increase in specificity up to 63% (AUC 0.84); in fact, the median values of estimated risk probability were significantly lower in patients with RP than in those with COVID-19 (*p* = 0.001). This result supports the idea that risk stratification rather than binary classification is helpful in discriminating different entities with overlapping CT features [16]. Moreover, these data reinforce the idea that deep learning algorithms based only on CT images cannot distinguish COVID-19 pneumonia from other lung interstitial diseases with overlapping CT features with high specificity; thus, adding clinical/laboratory findings to the algorithm can improve the diagnostic performance based on binary classification. In the case of the deep learning algorithm used in this study, CT scans classified as low risk (below the 30% threshold) matched with a positive molecular test in less than 5% of the cases (preliminary study in our institution). Nevertheless, further data collection is needed to improve the generalizability of our results. In addition, total lung volume involvement was significantly higher in COVID-19 than in RP patients, as an expected consequence of the typical bilateral and multi-lobar lung involvement of COVID-19. Moreover, the RP pattern is usually strictly related to the target volume and to the dose distribution of the treatment plan. The lobar involvement analysis demonstrated that both lower lobes were significantly less affected in patients with RP than in patients with COVID-19 pneumonia. These differences in chest CT patterns are the main factors that justify the cut-off value for suspected COVID-19 pneumonia and the good performance of the deep learning algorithm. The AI algorithm was able to detect COVID-19 pneumonia features on chest CT images of a tested population (Caucasian) with ethnic characteristics different from the population used for training (Asian). The algorithm showed good performance in segmentation of the most typical CT findings in COVID-19 pneumonia, such as multifocal and peripheral, often bilateral, ground-glass areas associated with patchy consolidations. The performance of the algorithm was excellent in terms of sensitivity and negative predictive value for the detection of COVID-19 pneumonia (AUC 0.99) and confirmed the utility of this software as a rapid diagnostic tool to flag suspected COVID-19 patients. This study has also some limitations: it is retrospective in nature, the size of the samples is small and the protocols used with three different scanners were variable. In addition, overlapping chest CT features of several diseases reflect common mechanisms of response of the lungs to different etiologies. Subsequently, measurements of volume, shape or density of pulmonary lesions may not be sufficient to develop powerful deep learning models. The study does, however, confirm that artificial intelligence solutions can assist in the clinical management and follow-up of patients with cancer in the COVID-19 pandemic and mitigation phases.

## 5. Conclusions

In conclusion, the deep learning algorithm used in this study is able to discriminate RP from COVID-19 pneumonia, classifying most RP cases as “COVID-19 Low Risk”. These results may be improved in time with the addition of clinical data, leading to more accurate AI solutions in the differential diagnosis of lung diseases with interstitial involvement. Our results suggest that lesion distribution as well as other radiomics-based data and clinical information are necessary to improve the reliability of AI algorithms for the diagnosis of radiation therapy-related pneumonitis.

## Figures and Tables

**Table 1:** Characteristics of the recruited samples.

Variable	Pneumonia-Free	COVID-19	RP
Patients (*n*)	30	34	36
Female/Male (*n*)	13/17	15/19	22/14
Age (Years)	59 (32–88)	67 (38–87)	72 (49–87)
SARS-CoV-2 RT-PCR (Positive/Negative/n.a.)	0/14/16	34/0/0	0/0/21
NSCLC/SCLC	0/0	0/0	32/4
Radiation Dose (Gy)	n.a.	n.a.	54 ± 6.7 Gy
AI Class: No COVID-19	30/30	1/34	1/36
AI Class: COVID-19 Low Risk	0/30	7/34	24/36
AI Class: COVID-19 High Risk	0/30	26/34	11/36

Age reported as median value (minimum and maximum). COVID-19 = coronavirus disease 2019; RP = radiation pneumonitis; SARS-CoV-2 = severe acute respiratory syndrome coronavirus 2; RT-PCR = reverse transcriptase polymerase chain reaction; AI = artificial intelligence; NSCLC = non-small cell lung cancer; n.a. = not applicable.

**Table 2:** Diagnostic performance of the deep learning algorithm across the different group comparisons.

Comparison	Sensitivity	Specificity	VPP	VPN	Accuracy	AUC
COVID-19 vs. pneumonia-free	97.1 (88.6–97.1)	100 (90.4–100)	100 (91.2, 100)	96.8(87.5, 96.8)	98.4%	0.99
COVID-19 vs. others	97% (0.85–0.99)	47% (0.4–0.48)	48% (0.42–0.49)	97% (0.84–0.99)	64%	0.85
COVID-19 vs. RP	97% (0.94–0.99)	2% (0–0.05)	48% (0.47–0.49)	50% (0.02–0.97)	48%	0.72
COVID-19 vs. RP (cut-off 30%)	76% (0.63–0.87)	63% (0.51–0.74)	67% (0.55–0.76)	96% (0.83–0.99)	70%	0.84

Values in parentheses are 95% CI. PPV = positive predictive value; NPV = negative predictive value; AUC = area under the receiver operating characteristic (ROC) curve; COVID-19 = coronavirus disease 2019; RP = radiation therapy-related pneumonitis.

**Table 3:** Comparison between COVID-19 pneumonia and radiation therapy-related pneumonitis (RP), based on total and lobar involvement.

		COVID-19	RP	*p*-Value
Total	(%)	2.95 (1.22–8.89)	0.51 (0.16–1.99)	0.001
	(cm^3^)	105.54 (44.68–257.07)	29.14 (5.59–69.20)	0.001
RUL	(%)	0.78 (0.15–5.12)	0.29 (0–1.59)	0.12
	(cm^3^)	7.3 (1.21–31.42)	2.05 (0.04–11.65)	0.052
ML	(%)	0.24 (0–3.89)	0 (0–0.59)	0.033
	(cm^3^)	1.01 (0–7.92)	0 (0–2.46)	0.045
RLL	(%)	3.54 (1.19–11.06)	0.15 (0–0.9)	<0.001
	(cm^3^)	27.14 (8.20–83.30)	1.3 (0–5.77)	<0.001
LUL	(%)	0.73 (0.05–5.70)	0.29 (0–1.59)	0.042
	(cm^3^)	7.22 (0.84–54.28)	0.98 (0–28.28)	0.032
LLL	(%)	3.99 (0.46–17.56)	0.005 (0–2.32)	<0.001
	(cm^3^)	16.35 (3.66–85.61)	0.06 (0–14.76)	<0.001
COVID-19 Risk (%)		41.85 (34.52–51.12)	27.35 (20.09–35.5)	0.001

Values are reported as median values of the relative percentage of lobar involvement and absolute volumes. Values in parentheses are 25% and 75% percentiles that were used instead of the minimum and maximum, as the value 0 was frequent in the distribution. COVID-19 = coronavirus disease 2019; RUL = right upper lobe; ML = middle lobe; RLL = right lower lobe; LUL = left upper lobe; LLL = left lower lobe.

## Data Availability

Raw data are readily available for presentation to the referees and the editors of the journal, if requested. The authors ensure that raw data is retained in full for at least 5 years after publication.
